# Effect of Maternal Factors on Problematic Smartphone Use among Elementary School Children

**DOI:** 10.3390/ijerph18179182

**Published:** 2021-08-31

**Authors:** Eun Jee Lee, Hee Sun Kim

**Affiliations:** College of Nursing, Research Institute of Nursing Science, Jeonbuk National University, Jeonju 54896, Korea; ejlee@jbnu.ac.kr

**Keywords:** problematic smartphone usage, maternal factors, elementary school students, parent–child communication, social network service, Korea

## Abstract

Mothers play a key role in ensuring their children’s healthy life. This study aimed to identify the maternal factors affecting problematic smartphone use in children. We adopted a cross-sectional descriptive design using structured questionnaires. Participants were fourth to sixth grade elementary school students in Korea (*n* = 184). The following maternal factors were evaluated: maternal mediation, children’s perception of mothers’ smartphone use, mother–child communication, and parenting style. Data regarding maternal factors’ effect on problematic smartphone use were analyzed by computing descriptive statistics and logistic regression analysis using SPSS Win 27.0. The results show that the maternal factors that influenced problematic smartphone use in children were maternal control over smartphone usage (odds ratio (OR) = 5.10, 95% confidence interval (CI): 1.33–19.60), smartphone usage time for social network service (OR = 1.52, 95% CI: 1.10–2.01), and problematic mother–child communication (OR = 1.07, 95% CI: 1.00–1.14). Therefore, to promote appropriate smartphone use among school children, it is necessary to develop an intervention comprising maternal supervision of their children’s smartphones, guidance provision for social network service usage, and strategies for the reinforcement of positive mother–child communication.

## 1. Introduction

Smartphones are indispensable devices within our modern society. They have several advantages, such as allowing novel idea and knowledge acquisition, social contact and support provision opportunities, and increased health information acquisition opportunities [1]. Korea reports one of the highest global smartphone penetration rates, with a proportion surge from 59.3% in 2015 to 81.2% in 2018 among school-age children [2].

The increased number of smartphone users has led to the emergence of various problems in school-age children, such as weight gain; poor sleep quality; exposure to inaccurate, inappropriate, or unsafe content; and threats of personal identity exposure [1,3,4,5]. Research from a psychological perspective suggests that children who overuse smartphones have poor mental health and may experience anxiety and depression [4,6]. Similarly, behavioral research demonstrates that children who overuse smartphones have fewer opportunities for social interaction than do normal children. This may result in a lack of social skills and emotional control. Therefore, these children may display aggression, hyperactivity, lying, etc. [4,7]. The Korea government conducts an annual survey on smartphone usage habits [6]. The survey results suggest that the rate of problematic smartphone use (PSU) continues to increase among the fourth and sixth graders of elementary school, while the lower age limit of problematic smartphone users is gradually lowering. Moreover, in 2019, the proportion of problematic smartphone users among elementary school students was on par with middle school students, who comprised the highest proportion of PSU addicts in Korea [8].

Parents tend to have a significant impact on the development of their children’s health and lifestyle [9]. Previous studies have identified parental factors that affect children’s problematic behaviors. Parenting and parent–child relationships are strongly related to early adolescent delinquency [10] and adolescents’ internet addiction [11], while parenting style is related to adolescents’ harmful behaviors such as tobacco or alcohol consumption [12]. In terms of parental impact on smartphone addiction, parental smartphone use is strongly associated with children’s PSU [13,14]. Due to the high influence of parents on their children in many ways, they struggle to manage their child’s smartphone use and to become positive role models regarding appropriate smartphone usage, such as by modeling their own smartphone use [15,16]. In addition, parents’ mediation played an important role in decreasing school-aged children’s PSU [17], and deteriorating parent–child communication was linked to higher smartphone addiction in girls aged 11–13 years [18].

Meanwhile, there may be gender differences in the approaches to problem-solving [19]. Therefore, children’s PSU may be differently impacted by mothers and fathers. However, mothers have a relatively higher impact on their children. Of both the parents, children’s problematic behaviors are highly influenced by their mothers’ various conditions. Mothers’ smartphone addiction can influence early smartphone exposure in school-aged children [16]. Restrictive maternal mediation, perceived as privacy invasion, is related to internet addiction in adolescents [20]. In addition, improved mother–child relationship quality reduced problematic behaviors such as substance use in children aged 12 years [21], and deteriorating mother–child communication influenced smoking behaviors in adolescents [22]. Moreover, mothers’ mental health influenced their parenting style toward their children [23], and preschool children raised by mothers with negative parenting styles demonstrated higher behavioral problems and poor prosocial functioning during the children’s first grade [24]. Thus, based on previous studies, maternal factors are considered important for PSU among children and adolescents. Furthermore, in Korea, school age is a marker for owning one’s first smartphone [2], and thus, it is a critical period for the development of appropriate smartphone usage habits. However, research on the relationships between elementary school children’s PSU and maternal factors is still limited. Therefore, this study aimed to investigate the effect of maternal factors (mother’s mediation, mother’s smartphone use, mother–child communication, and parenting style) on school children’s PSU and to provide basic data for developing interventions for preventing children’s PSU through parental participation.

## 2. Methods

### 2.1. Design

This study adopted a cross-sectional descriptive design using structured questionnaires to identify the maternal factors that influence PSU in children.

### 2.2. Participants and Data Collection

For this survey, we recruited elementary school students, aged 10–12 years (grades 4–6), including those who owned smartphones and had no problems in reading, understanding, and writing Korean. Participants in this study were limited to children who owned smartphones, as these children are at a higher risk of PSU [17]. Currently, the South Korean education system mandates fundamental education comprising 6, 3, and 3 years of elementary (grades 1–6), middle (grades 7–9), and high school (grades 10–12), respectively. Furthermore, students appear for a national entrance test for universities after graduating high school.

A pilot survey was conducted using the questionnaire draft among 10 grades 4–6 elementary school students to determine the item difficulty. The power analysis using G*Power (3.1.9.2) indicated a minimal sample size of 147 participants to perform a logistic regression with 80% statistical power (effect size = 0.55, α = 0.05). Assuming a smartphone penetration and questionnaire response rate of 70% each, we planned this survey for about 300 participants. A research assistant visited the teachers of three elementary schools to collect the data and explained the purpose and process of the research. The data were collected in December 2020 using a structured questionnaire. Questionnaires were collected from 327 participants, and 291 (89.0%) of them owned a smartphone. Of these, 107 questionnaires were excluded due to missing data on at least one item, and the remaining 184 (63.2%) questionnaires were analyzed. We performed a Little’s Missing Completely at Random test using SPSS, and results indicated that the missing data in this study were completely random (χ^2^ = 7709.70, *p* = 0.435).

### 2.3. Measures

#### 2.3.1. Problematic Smartphone Use

PSU was evaluated using the Smartphone Addiction Proneness Scale (SAPS). This tool has demonstrated good reliability and validity [25], and it is employed in annual public surveys to gather basic data for policy-making for Korean children and adolescents. The SAPS comprises 15 items in four subdomains: disturbance of adaptive functions (5 items), virtual life orientation (2 items), withdrawal (4 items), and tolerance (4 items). Each item was scored on a 4-point Likert-type response scale (1 = strongly disagree, 4 = strongly agree) [25]. The SAPS categorizes scorers depending on the PSU level into three groups: ≤41 (normal users), 42–44 (at-risk users), and ≥45 (high-risk users). In this study, we grouped the at-risk and high-risk users into the risk group, while normal users were categorized as the normal group. This scale reported high inter-item consistency, with a Cronbach’s alpha of 0.88 at the time of development [25] and 0.85 in this study.

#### 2.3.2. Maternal Mediation

The Parental Mediation of Children’s Internet Use Tool [26] was used to measure maternal mediation of children’s smartphone usage, after being translated and modified into Korean [18]. The instrument comprises 24 items with five subdomains: active use mediation (5 items), active safety mediation (6 items), monitoring (4 items), technical mediation (3 items), and restrictive mediation (6 items). Each item was scored on a 2-point response scale (0 = no, 1 = yes). Higher scores indicated the higher level of maternal mediation. This scale reported high inter-item consistency, with a Cronbach’s alpha of 0.80 at the time of adaptation into Korean [18] and 0.71 in this study.

#### 2.3.3. Perception of Mother’s Smartphone Use

The perception of mothers’ smartphone use was measured using Stockdale’s Technoference tool [13]. Technoference refers to the interference, intrusion, or interruption of social interaction due to social media or technology. In this study, it specifically refers to the interruption or abrupt termination of mothers’ communication upon receiving a call or message on their smartphone. The researchers translated Stockdale’s instrument [13] to Korean, and content validity was verified by two experts. It comprises three items with a 5-point Likert-type response scale (1 = not at all, 5 = a great deal). Higher scores indicated higher levels of technoference. This scale reported high inter-item consistency, with a Cronbach’s alpha of 0.85 at the time of development [13] and 0.66 in this study.

#### 2.3.4. Mother–Child Communication

The Parent-Adolescent Communication Scale (PACS) [27] was used to evaluate mother–child communication. PACS was translated and adapted into Korean, and content validity was verified in Kim’s (1990) study [28]. It comprises 20 items in two subdomains: openness (10 items) and problems (10 items). Each item was scored on a 5-point Likert-type response scale (1 = strongly disagree, 5 = strongly agree). Higher scores on the openness subdomain indicated more open and positive mother–child communication, while higher scores on the problems subdomain indicated more negative and hindered mother–child communication. This scale reported high inter-item consistency, with a Cronbach’s alpha of 0.88 (openness) and 0.78 (problems) at the time of adaptation into Korean [28] and 0.88 (openness) and 0.84 (problems) in this study.

#### 2.3.5. Parenting Style

The Inventory for Parenting Behavior Perceived by Adolescents [29] was used to evaluate parenting style. This tool has demonstrated good reliability and validity [29], and it is employed in annual public surveys to gather basic data for policy-making for Korean children and adolescents. The parenting style measure comprises 32 items in six subdomains: monitoring (4 items), reasoning (5 items), inconsistency (4 items), over-expectation (6 items), intrusiveness (7 items), and affection (6 items). Each item was scored on a 4-point Likert-type response scale (1 = strongly disagree, 4 = strongly agree) [29]. Higher scores for a subdomain indicated stronger tendencies toward that subdomain. This scale reported high inter-item consistency, with a Cronbach’s alpha of 0.75–0.83 at the time of development [29] and 0.67–0.89 in this study.

#### 2.3.6. General Characteristics

Participants provided information about gender, grade level, perceived maternal control (single item: “How much do you think your parents supervise your smartphone use?”), age, age of owning first smartphone, smartphone usage duration (weekdays and weekend), and smartphone usage duration by purpose.

### 2.4. Data Analysis

Data were analyzed using the SPSS WIN 27.0 program. Demographic information was examined using descriptive statistics. Chi-squared test and independent *t*-test were used to compare the differences in general characteristics and research variables between the normal and the risk group participants. Spearman correlation analysis was employed to investigate the association between research variables. Furthermore, PSU-related maternal factors were identified using binary logistic regression analysis. Statistical significance was set at *p* < 0.05.

### 2.5. Ethics Consideration

This study was approved by the Ethics Committee of the Jeonbuk National University Hospital (JBNU 2020-12-010-001). Participants and their parents were informed about the purpose and procedures of this study and were provided with written consent form. Furthermore, the participants were provided with written consent forms. It was emphasized that data collection was anonymous, and data would be kept confidential. Participants were also informed that they could withdraw from the study at any time without any consequences. Accordingly, participants completed the questionnaire after providing informed consent.

## 3. Results

### 3.1. Difference in General and Smartphone Use Characteristics According to Normal and Risk Group of PSU

Table 1 presents the general participant characteristics categorized into normal and risk groups for PSU. The PSU risk group comprised 17.9% of the total sample. The results show significant differences in perceived maternal control, smartphone usage duration (weekdays and weekend), and smartphone usage duration by purpose. The normal group perceived that their parents occasionally or frequently control their smartphone usage (χ^2^ = 9.16, *p* = 0.010). The risk group reported higher smartphone usage duration on weekdays (*t* = −4.61, *p* < 0.001) and weekends (*t* = −4.50, *p* < 0.001), and for accessing social network services (SNS; *t* = −3.28, *p* = 0.002) and video clips (*t* = −2.66, *p* = 0.011).

### 3.2. Comparing Maternal Factors between Normal and Risk PSU Groups

Table 2 presents the differences in research variables between the normal and risk PSU groups. The risk group reported significantly higher scores for disturbance of adaptive functions (*t* = −11.05, *p* < 0.001), withdrawal (*t* = −5.46, *p* < 0.001), tolerance (*t* = −10.38, *p* < 0.001) in PSU subdomains; problematic mother–child communication (*t* = −3.44, *p* = 0.001); and for inconsistent (*t* = −2.31, *p* = 0.022) and affectionate parenting style (*t* = 2.51, *p* = 0.013) than the normal group.

### 3.3. Relationship between Research Variables

Table 3 presents the relationships between the variables of school-aged children. The results demonstrate significant correlations of PSU with SNS (*r* = 0.27, *p* < 0.001), video clips (*r* = 0.23, *p* < 0.001), technical maternal mediation (*r* = −0.15, *p* < 0.05), problematic mother–child communication (*r* = 0.23, *p* < 0.001), and inconsistent (*r* = 0.19, *p* < 0.05) and affectionate parenting style (*r* = −0.17, *p* < 0.05).

### 3.4. Multivariate Binary Logistic Regression for PSU-Related Maternal Factors in Elementary School Students

Table 4 presents the results of the multivariate binary logistic regression models. The following maternal factors were significantly associated with PSU in elementary school students (*n* = 184): children’s perception that their parents control hardly their smartphone usage (odds ratio (OR): 5.10, confidence interval (CI): 1.33–19.60), longer smartphone usage duration for accessing SNS (OR: 1.52, CI: 1.10–2.01), and problematic mother–child communication (OR: 1.07, CI: 1.00–1.14).

## 4. Discussion

This study aimed to provide basic data for developing PSU prevention intervention programs through parent participation by identifying the maternal factors that influence PSU in school children. The results suggest that the maternal factors of maternal control over children’s smartphone usage, smartphone usage for SNS, and problematic mother–child communication impact PSU.

In this study, the PSU risk group comprised 17.9% of the total sample. According to the Korean national statistics, the proportion of PSU users was 17.1%, which is slightly lower than in this study [14]. In this study, data were collected in December 2020, and during this period children had been attending virtual classes from home for nearly a year due to the COVID-19 pandemic. These circumstances have resulted in an increase in the proportion of smartphone addicts worldwide and in Korea [14,30]. Therefore, it is necessary to monitor the PSU rate for children for a while. In addition, various measures are needed to shape appropriate smartphone usage habits among children in this COVID era.

In this study, the tolerance subdomain reported the highest scores among the four SAPS subdomains in PSU risk group. Furthermore, the PSU risk group demonstrated the most difficulty in reducing smartphone usage duration. Individuals with unsatisfied psychological needs tend to act toward ignoring or alleviating them [31]. Thus, children’s increasing use of smartphones may aim to satisfy unmet psychological needs. A meta-analysis revealed that higher levels of depression, anxiety, and perceived stress were associated with PSU among children and adolescents [32]. Another study found that low self-control and self-esteem may increase the risk of PSU, while academic achievement might lower this risk in children [33]. Meanwhile, Korean parents place high emphasis on education and tend to ignore their children’s unmet psychological desires amidst academic pursuits [34]. Therefore, it is necessary to evaluate and mitigate children’s unfulfilled needs to ensure appropriate smartphone usage among them and develop psychological interventions (e.g., cognitive behavior therapy) for smartphone addiction among elementary school students.

Mothers have a major influence on their children’s lifestyles [24]. In this study, perceived maternal control, which indicated children’s perception regarding the degree of maternal supervision of their smartphone use, affected children’s PSU. Furthermore, it was found that 8.1% of the children perceived that their mothers did not supervise their smartphone use at all, and these children were 5.1 times more likely to be at-risk than those who perceived that their mothers frequently or almost always supervised their smartphone usage. Previous studies suggest that parental control imbues appropriate smartphone usage among children and reduces their impulsiveness [15]. The American Association of Pediatrics suggested that it is necessary for parents to supervise their children’s smartphone use to ensure proper smartphone use [1]. Therefore, parents who did not monitor their children’s smartphone usage must understand the need for supervision and subsequently educate themselves about good supervision methods.

Parental mediation refers to the intervention strategies that parents employ to guide children’s appropriate smartphone usage and minimize its negative consequences [35]. In this study, maternal mediation did not affect children’s PSU, which is inconsistent with previous study findings [17,36,37]. However, maternal mediation was significantly correlated with smartphone usage time by purpose, parent–child communication, and parenting style. Thus, there may be a possibility of an indirect effect of maternal mediation on children’s PSU. Therefore, longitudinal studies are needed to determine the long-term effects of maternal mediation on children’s PSU. Another possible explanation may be that this study investigated children’s perceptions of maternal mediation strategies, rather than obtaining primary data from the mother. Accordingly, we recommend that future researchers investigate mothers’ smartphone use and mediation directly.

Communication impacts children’s problematic behavior [10]. It is necessary to evaluate and mitigate children’s unfulfilled needs in any case, regardless of their use of smartphones. Mothers should also be educated about how to prevent problematic communication regardless of the use of smartphones by children. This study revealed that problematic maternal communication affected children’s PSU. Another previous study suggested that the quantity and quality of family conversations affected individual PSU [38]. In addition, adolescents who spend significant amounts of time talking with their mothers are more likely to avoid PSU [39]. The current study results indicate that avoiding negative mother–child communication is important to ensure children’s correct smartphone use. Therefore, mothers should be educated to prevent problematic communication and reduce the risk of PSU in children. Moreover, they should acquire efficient communication skills to avoid adverse effects on the mother–child relationship when they monitor their children’s smartphone usage.

In this study, mothers’ perceived smartphone use and parenting style did not affect PSU. Several studies have demonstrated the adverse effect of PSU on interpersonal relationships [4,7,14]. According to existing literature, children’s perception of their parents’ problematic smartphone use may result in parent–child conflict and affect their relationship. However, if a mother’s smartphone use is not perceived as a problem, it does not cause conflict, and smartphone use can replace other shared activities between mothers and children [40,41]. Therefore, in this study, children’s perception of their mothers’ smartphone use may not affect their PSU. Therefore, additional research is needed to determine the changes in parent–child relationships due to children’s perceptions of their mother’s smartphone use. In a previous study, negative parenting styles resulted in higher behavioral problems [24]. In this study, the mother’s parenting attitude did not affect the child’s PSU directly, but the parenting style subdomains of inconsistency and affection were significantly correlated with children’s PSU. Therefore, it is necessary for mothers to maintain a consistent and affectionate parenting attitude while implementing maternal interventions.

The risk group in this study reported significantly longer smartphone use duration for SNS. SNS is advantageous for personal interaction through the creation of online identities, communication with others, and formation of social networks [42]. A previous study reported that SNS is a health-related information tool, and it also evokes a strong feeling of social belonging among people who feel rejected [43]. Ironically, these factors also increase the addiction to SNS [17].

Watching video clips did not affect PSU directly. However, risk users spent significantly longer hours watching video clips than normal users. Moreover, video clip watching has been increasing in both Korea and the United States [1,44]. Watching video clips (e.g., YouTube) is overused because it provides gratification of four types: hedonic, affection, information, and social [45]. Additionally, SNS usage is majorly similar to watching video clips, except the latter is accompanied by the acts of sharing, posting, and commenting [45]. Application developers implement various addictive software features, such as variable rewards, social reciprocity, infinite scrolling, illusion of choice, user investment, and gamification to obtain people’s attention [31,46]. Therefore, it is necessary to continuously monitor smartphone usage and establish rules through mutual agreement with children to ensure safe usage of SNS and video clip watching. Accordingly, software developers should ethically organize their programs with appropriate regulations, such as minimum age limits, which can be strictly followed.

This study has several limitations. First, this study employed convenience sampling methods in Korea; thus, the findings may not be generalizable to all school-aged children. Second, this study only investigated maternal factors. However, it is also essential to investigate the and role and influence of paternal factors on children’s lifestyle and health in future research. Last, we did not control for students’ demographic factors (parents’ marital status, family structure, mother’s education, and economic status). Future studies need to investigate the effect of maternal factors on children’s PSU after controlling for the child’s demographic factors.

## 5. Conclusions

This study aimed to provide basic data for developing preventive interventions for children’s PSU through maternal participation by identifying maternal factors that affect PSU in school-age children. The current study findings revealed that the maternal factor of monitoring children’s smartphone use, SNS usage and watching video clips, and mother–child communication influenced children’s PSU. Therefore, mothers must supervise their children’s smartphone usage to ensure appropriate use, and maternal guidance should be provided for social media usage and watching video clips. In addition, mothers should carefully facilitate positive communication with their children throughout this process. The current study findings can guide mothers’ participation in developing preventive interventions for children’s PSU. In addition, policies should be implemented to protect children from the negative effects of smartphone content use.

## Figures and Tables

**Table 1:** Differences in general and smartphone characteristics according to normal and risk group of PSU (*n* = 184).

Variables	Categories	Total (*n* = 184)		Normal Group (*n* = 151, 82.1%)		Risk Group (*n* = 33, 17.9%)		χ^2^ or *t*	*p*
		N (%) or Mean (SD)		N (%) or Mean (SD)		N (%) or Mean (SD)			
Gender	Boy	82	(44.6)	71	(86.6)	11	(13.4)	2.05	0.152
	Girl	102	(55.4)	80	(78.4)	22	(21.6)		
Grade	4th	34	(18.5)	30	(88.2)	4	(11.8)	1.11	0.576
	5th	55	(29.9)	44	(80.0)	11	(20.0)		
	6th	95	(51.6)	77	(81.1)	18	(19.0)		
Perceived maternal control	Hardly ever	15	(8.1)	8	(53.3)	7	(46.7)	9.16	0.010
	Occasionally	103	(56.0)	87	(84.5)	16	(15.5)		
	Frequently	66	(35.9)	56	(82.1)	10	(15.2)		
Age (years)		11.34	(0.88)	11.31	(0.88)	11.45	(0.91)	−0.84	0.401
Age of owning first smartphone (years)		9.92	(1.55)	9.98	(1.55)	9.67	(1.56)	1.05	0.293
Smartphone usage duration by period (hours)	Weekdays	3.10	(2.31)	2.66	(1.91)	5.10	(2.91)	−4.61	<0.001
	Weekends	4.93	(3.42)	4.28	(2.79)	7.87	(4.40)	−4.50	<0.001
Smartphone usage time by purpose (hours/day)	Online games	1.32	(1.29)	1.27	(1.27)	1.52	(1.37)	−0.99	0.326
	SNS	0.92	(1.22)	0.74	(1.03)	1.73	(1.66)	−3.28	0.002
	Chatting	1.05	(1.22)	0.94	(1.01)	1.59	(1.83)	−1.98	0.056
	Video clips	2.24	(2.14)	2.01	(1.98)	3.28	(2.58)	−2.66	0.011

PSU: problematic smartphone use, SNS: social network service, SD: standard deviation.

**Table 2:** The Difference in descriptive statistics of the study variables according to PSU (*n* = 184).

Variables	Normal Group (*n* = 151, 82.1%)		Risk Group (*n* = 33, 17.9%)		*t*	*p*
	Mean (SD)		Mean (SD)			
PSU						
Disturbance of adaptive functions	1.95	(0.41)	2.83	(0.42)	−11.05	<0.001
Virtual life orientation	2.48	(0.46)	2.45	(0.59)	0.26	0.793
Withdrawal	1.66	(0.47)	2.42	(0.77)	−5.46	<0.001
Tolerance	2.12	(0.54)	3.18	(0.52)	−10.38	<0.001
Maternal mediation						
Active use mediation (range 0–5)	2.11	(1.46)	2.03	(1.49)	0.29	0.770
Active safety mediation (range 0–6)	3.09	(2.06)	2.58	(2.12)	1.29	0.201
Monitoring (range 0–4)	0.80	(1.11)	0.52	(0.94)	1.35	0.180
Technical mediation (range 0–3)	0.89	(1.11)	0.52	(1.00)	1.81	0.072
Restrictive mediation (range 0–6)	0.91	(1.07)	0.73	(1.07)	0.91	0.370
Perception of mother’s smartphone use	6.78	(2.29)	7.42	(2.21)	−1.47	0.143
Mother–child communication						
Openness	35.73	(7.44)	33.94	(8.33)	1.23	0.222
Problems	21.38	(6.77)	25.97	(7.69)	−3.44	0.001
Parenting style						
Monitoring	12.94	(2.20)	12.06	(2.95)	1.62	0.113
Reasoning	14.97	(3.15)	14.36	(3.03)	1.01	0.312
Inconsistency	8.76	(2.57)	9.88	(2.37)	−2.31	0.022
Over-expectation	11.59	(3.96)	12.76	(3.74)	−1.55	0.123
Intrusiveness	14.40	(4.45)	15.76	(4.55)	−1.58	0.115
Affection	20.38	(3.31)	18.70	(4.19)	2.51	0.013

PSU: problematic smartphone use, SD: standard deviation. Explanation of bold.

**Table 3:** Spearman correlation analysis among research variables (*n* = 184).

Variables			(1) PSU	(2)	(3)	(4)	(5)	(6)	(7)	(8)	(9)	(10)	(11)	(12)	(13)	(14)	(15)	(16)	(17)	(18)	(19)
(2)	Age of owning first smartphone		0.07																		
(3)	Smartphone usage time by purpose	Online games	0.08	−0.05																	
(4)		SNS	0.27 **	0.26 **	0.22 **																
(5)		Chatting	0.14	0.29 **	0.13	0.53 **															
(6)		Videoclips	0.23 **	0.24 **	0.32 **	0.27 **	0.37 **														
(7)	Maternal mediation	Active use mediation	−0.02	0.08	−0.09	0.00	0.09	−0.05													
(8)		Active safety mediation	−0.10	−0.14	−0.04	0.06	0.08	−0.13	0.39 **												
(9)		Monitoring	−0.11	−0.12	−0.08	−0.10	−0.13	−0.15 *	0.33 **	0.28 **											
(10)		Technical mediation	−0.15 *	−0.05	−0.17 *	−0.17 *	−0.16 *	−0.24 **	0.09	0.07	0.49 **										
(11)		Restrictive mediation	−0.09	−0.21 **	−0.04	−0.13	−0.08	−0.09	−0.01	0.18 *	0.24 **	0.09									
(12)	Perception of mother’s smartphone use		0.12	0.08	0.13	0.16 *	0.05	0.15 *	−0.04	−0.07	−0.07	−0.03	−0.12								
(13)	Mother–child communication	Openness	−0.09	−0.01	0.02	−0.03	0.09	−0.12	0.32 **	0.38 **	0.09	−0.06	0.13	−0.22 **							
(14)		Problems	0.23 **	0.14	0.04	0.18 *	0.00	0.12	−0.05	−0.23 **	0.05	0.03	−0.11	0.32 **	−0.59 **						
(15)	Parenting style	Monitoring	−0.12	−0.09	−0.16 *	−0.25 **	−0.15 *	−0.11	0.21 **	0.19 **	0.18 *	0.12	0.21 **	−0.06	0.40 **	−0.32 **					
(16)		Reasoning	−0.08	−0.05	−0.02	0.04	0.10	−0.01	0.23 **	0.43 **	0.07	−0.03	0.11	−0.17 *	0.53 **	−0.42 **	0.40 **				
(17)		Inconsistency	0.19 *	0.05	0.10	0.12	−0.05	0.06	−0.05	−0.08	0.04	−0.01	−0.10	0.25 **	−0.37 **	0.64 **	−0.22 **	−0.37 **			
(18)		Over-expectation	0.13	0.06	0.18 *	0.09	−0.07	−0.05	0.02	−0.01	0.11	0.07	−0.10	0.03	−0.15 *	0.33 **	−0.17 *	−0.11	0.47 **		
(19)		Intrusiveness	0.14	0.11	0.05	0.13	−0.07	0.02	0.04	−0.07	0.09	0.04	−0.05	0.28 **	−0.44 **	0.66 **	−0.21 **	−0.34 **	0.56 **	0.45 **	
(20)		Affection	−0.17 *	−0.06	0.02	−0.02	0.11	−0.07	0.19 **	0.37 **	0.02	−0.05	0.13	−0.25 **	0.68 **	−0.61 **	0.36 **	0.59 **	−0.37 **	−0.22 **	−0.51 **

PSU: problematic smartphone use; ** *p* < 0.001, * *p* < 0.05 (two-tailed).

**Table 4:** Maternal factors affecting PSU in school-age children.

Variables	Categories	OR	(95% CI)	*p*
Perceived maternal control	Hardly ever (vs. Frequently)	5.10	(1.33,19.60)	0.018
Smartphone usage time by purpose (hours/day)	SNS	1.52	(1.10, 2.01)	0.011
Mother–child communication	Problems	1.07	(1.00, 1.14)	0.035

PSU: problematic smartphone use, SNS: social network service, OR: odds ratio, CI: confidence interval.

## Data Availability

The data used and/or analyzed during the current study are available from the author on request.
